# Sulfation of Wheat Straw Soda Lignin with Sulfamic Acid over Solid Catalysts

**DOI:** 10.3390/polym14153000

**Published:** 2022-07-25

**Authors:** Aleksandr S. Kazachenko, Feride Akman, Natalya Yu. Vasilieva, Yuriy N. Malyar, Olga Yu. Fetisova, Maxim A. Lutoshkin, Yaroslava D. Berezhnaya, Angelina V. Miroshnikova, Noureddine Issaoui, Zhouyang Xiang

**Affiliations:** 1 Institute of Chemistry and Chemical Technology, Krasnoyarsk Scientific Center, Siberian Branch, Russian Academy of Sciences, Akademgorodok 50, Bld. 24, 660036 Krasnoyarsk, Russia; vasilyeva.nata@mail.ru (N.Y.V.); yumalyar@gmail.com (Y.N.M.); fou1978@mail.ru (O.Y.F.); maximsfu@yahoo.com (M.A.L.); zyppa90298@gmail.com (Y.D.B.); miroshnikova35@gmail.com (A.V.M.); 2 School of Non-Ferrous Metals and Materials Science, Siberian Federal University, pr. Svobodny 79, 660041 Krasnoyarsk, Russia; 3 Vocational School of Food, Agriculture and Livestock, University of Bingöl, Bingöl 12000, Turkey; chemakman@gmail.com; 4 Laboratory of Quantum and Statistical Physics (LR18ES18), Faculty of Sciences, University of Monastir, Monastir 5079, Tunisia; issaoui_noureddine@yahoo.fr; 5 State Key Laboratory of Pulp and Paper Engineering, South China University of Technology, Guangzhou 510640, China; fezyxiang@scut.edu.cn

**Keywords:** lignin, sulfation, catalysts, sulfated lignin, DFT

## Abstract

Soda lignin is a by-product of the soda process for producing cellulose from grassy raw materials. Since a method for the industrial processing of lignin of this type is still lacking, several research teams have been working on solving this problem. We first propose a modification of soda lignin with sulfamic acid over solid catalysts. As solid catalysts for lignin sulfation, modified carbon catalysts (with acid sites) and titanium and aluminum oxides have been used. In the elemental analysis, it is shown that the maximum sulfur content (16.5 wt%) was obtained with the Sibunit-4^®^ catalyst oxidized at 400 °C. The incorporation of a sulfate group has been proven by the elemental analysis and Fourier-transform infrared spectroscopy. The molecular weight distribution has been examined by gel permeation chromatography. It has been demonstrated that the solid catalysts used in the sulfation process causes hydrolysis reactions and reduces the molecular weight and polydispersity index. It has been established by the thermal analysis that sulfated lignin is thermally stabile at temperatures of up to 200 °C. According to the atomic force microscopy data, the surface of the investigated film consists of particles with an average size of 50 nm. The characteristics of the initial and sulfated β-O-4 lignin model compounds have been calculated and recorded using the density functional theory.

## 1. Introduction

Lignocellulosic biomass is known to be the most promising renewable carbon source on Earth [1,2]. The lignin fraction in lignocellulosic biomass ranges from 10–35%, depending on the biomass’ origin and type. In particular, hardwoods contain 20–30% lignin, conifers can contain 25–38%, agro-industrial wastes can contain 11–20%, and herbaceous plants can contain 8–15% [1]. The lignin from woody biomasses is an aromatic polymer consisting of phenylpropane units built from organic substituents, including ether, carboxyl, and alcohol groups, and the composition and amount of which depend on the initial biomass and the isolation method used [3].

The unique structure of lignin makes it a good candidate for chemical processing into valuable products, for example, in the production of phenolic and aromatic chemicals and liquid fuels [1,2,3,4,5]; however, its processing techniques are underdeveloped, in contrast to the case of polysaccharides.

Lignin attracts researches because of its rich aromatic composition (the high carbon content) and high biodegradability and thermal stability [1,2,3,6].

The lignin processing techniques involve widespread catalytic methods, both oxidative [7] and reductive [8]. Its chemical modification involves various categories, e.g., the depolymerization and fragmentation of lignin, modification via the synthesis of new reactive centers, the chemical modification of the hydroxyl groups, and the formation of lignin–graft copolymers [1,2,3].

Lignin finds industrial and biomedical applications in biofuels, chemicals, polymers, and drug delivery nanomaterials; these applications depend on the source, chemical modification, and physicochemical properties of lignin [1,2,3,6].

The industrial processing of lignin is complicated by its nature and strong structural and functional heterogeneity predetermined by a chosen raw material and production conditions [9].

A promising application in the field of lignin processing is the production of lignin derivatives containing sulfate groups. This process makes the plant polymer water-soluble and increases its biodegradability. The sulfated lignin derivatives can not only replace the common products of the chemical modification of polysaccharides but also finds applications in pharmaceuticals as a potential new class of antiviral drugs and anticoagulants [6,10,11,12,13,14,15].

The available methods of the sulfation of carbohydrates, lignin, and related compounds are divided into two main groups. First, there are the conventional methods [6,10,11,12,13,14,15,16] that use aggressive sulfating reagents (sulfuric and chlorosulfonic acid, oleum, and sulfuric anhydride and its complexes with pyridine and toxic amines). These sulfating agents can sulfate not only aliphatic hydroxyl groups, but also phenolic hydroxyls, and thereby significantly degrade the biopolymer. An alternative to the above-listed reagents is sulfamic acid, a stable, low-toxic, non-hygroscopic, and noncorrosive crystalline substance obtained industrially based on the interaction of urea with oleum [17]. The sulfation of natural compounds with sulfamic acid can be carried out in both organic solvents and its eutectic with urea without an external solvent [18,19,20].

In [21,22,23], the catalytic effect of some organic bases on the sulfation of natural compounds with sulfamic acid was studied. Urea was shown to have the highest activity among the investigated bases [24,25,26,27,28].

A serious drawback of the available techniques of lignin sulfation with sulfamic acid in the presence of urea is the inability of recycling and catalyst reuse.

Currently, herbaceous lignins, including some agricultural straw types, are almost unused since these biopolymers have been understudied. The behavior of lignins during the chemical processing of plant raw materials is known to be determined by their structure; therefore, the structural arrangement of lignins and various botanical products of their modification should be thoroughly investigated. An enhanced knowledge of the structure of these polymers would expand the range of products obtained from plants [29]. It is well known that the characteristics of lignins depend on both the plant material type (species, geographic location, etc.) and the nature of the delignification process. Grass straw lignins were shown to be strongly different from tree-species lignins [30,31].

The aim of this study was to explore the effect of a type of solid acid catalyst on the sulfation of wheat straw soda lignin (WSSL) with sulfamic acid in a 1,4-dioxane medium, to elucidate the possibility of recycling, and to examine the composition and structure of the obtained products by Fourier-transform infrared and ultra-violet spectroscopy, gel permeation chromatography, density functional theory, and elemental analysis.

## 2. Materials and Methods

The reagents used were sulfamic acid and 1,4-dioxane produced by Khimreaktivsnab (Ufa, Russia) and Sibunit-4, TiO_2_, and γ-Al_2_O_3_ catalysts produced by Sigma-Aldrich (Saint Louis, MO, USA).

The oxidized carbon samples were prepared from a Sibunit-4 (S4) commercial mesoporous carbon material by oxidization in a mixture of 20 vol% of oxygen in N_2_ in the presence of water vapors at temperatures of 400 and 500 °C for 2 h [32]. The catalyst fractions were 1.0–1.6 mm (granular) and 0.056–0.094 mm (nongranular).

Sulfated Sibunit^®^ was obtained by the treatment of the graphite-like Sibunit^®^ material with sulfuric acid in the presence of nitric acid as described in [33,34].

The catalyst’s acidity was estimated using the point of zero charge (PZC) by the Sørensen–de Bruijn method [35,36], i.e., by measuring the acidity of a solid suspension upon reaching the isoelectric point (non-measurable electrode values) corresponding to pH of the solid suspension.

Sulfation of WSSL with sulfamic acid was carried out in a dioxane medium according to the modified procedure proposed in [27]. A total of 2 g of WSSL, 5 g of sulfamic acid (or ammonium sulfamate), and 50 mL of 1,4-dioxane were loaded into a heat-resistant three-necked flask with a capacity of 250 mL and placed in a thermostat with a mechanical stirrer; then, the mixture was heated to 90 °C under continuous stirring. Upon reaching the desired temperature, the reaction mixture was added with 0.5 g of a catalyst (see Table 1) and this temperature was held for 3 h. After that, the reaction mixture was cooled down to room temperature, the solvent was decanted, the residue was neutralized with a 5–7% aqueous ammonia solution to pH ~8–9 (50 mL), and a solid catalyst was filtered off. The WSSL aqueous solution was purified from low molecular weight compounds by dialysis carried out in an MF-503-46 MFPI cellophane dialysis bag (US) with a pore size of 3.5 kDa for 8–10 h, changing the water every hour. Next, the aqueous solution of sulfated lignin was evaporated to dryness under vacuum with a rotary evaporator and sulfated lignin in the form of an ammonium salt was obtained as a solid residue.

The elemental analysis of the sulfated samples was carried out on a Thermo Quest Flash EA-1112 elemental analyzer (Rome, Italy).

The Fourier-transform infrared (FTIR) spectra were recorded on a Shimadzu IRTracer-100 FTIR spectrophotometer (Kyoto, Japan) in the range of 4000–400 cm^−1^. The spectral data were processed using the LabSolution IR software. Solid samples for the FTIR spectroscopy study were tablets of the substance in the potassium bromide matrix prepared under the same conditions (i.e., the same time of mixing with potassium bromide, pressure, and evacuation time). The substance concentration was 3 mg per 1000 mg of KBr.

The ultraviolet-visible (UV-Vis) spectra were recorded on a Leki Instruments SS2109-UV scanning spectrophotometer (Lempäälä, Finland) using 1-cm quartz cells. The cell thermostating (±0.1 K) was performed by a Haake K15 thermostat connected to a Haake DC10 controller. The measured absorbance of the process solutions was 220–400 nm. All the measurements were performed at 298 K.

The weight average molecular weight *M*_w_, number average molecular weight *M*_n_, and polydispersity index PDI of the WSSL samples were determined by gel permeation chromatography (GPC) on an Agilent 1260 Infinity II multidetector GPC/SEC system with a refractive detector, viscometer, and light scattering detectors. The water-soluble samples were separated on two Agilent PL aquagel-OH Mixed-M columns using the aqueous solution of 0.1 M LiNO_3_ as a mobile phase. The column was calibrated using Agilent polyethylene glycol standards (US). The tetrahydrofuran-soluble samples were separated on an Agilent PLgel Mixed-B column with tetrahydrofuran stabilized with 250 ppm of butylhydroxytoluene (BHT) as a mobile phase. The column was calibrated using Agilent polystyrene standards (US). The eluent flow rate was 1 mL/min and the sample volume was 100 μL. Before the analysis, the samples were dissolved in the mobile phase (~5 mg/mL) and filtered through a 0.22-μm Agilent PTFE membrane filter. The data collection and processing were performed with the Agilent GPC/SEC MDS software.

The thermal analysis included the thermogravimetry (TG) and differential thermogravimetry (DTG) investigations and was carried out on a NETZSCH STA 449 F1 Jupiter synchronous thermal analysis instrument. Thermograms were taken in a corundum crucible in the argon atmosphere at temperatures of 30–800 °C and a sample heating rate of 10 °C/min.

### Calculation Details

In the calculation, the Gaussian 09W [37] and GaussView 0.5 [38] program packages were used. The molecular structure of the initial and sulfated β-O-4 lignin were optimized using the density functional theory (DFT) with the 6-31G(d, p) basis set via the Becke-3-parameter-Lee–Yang–Parr (B3LYP) hybrid functional levels [39,40]. The vibrational frequencies were calculated at the same level of theory; the determined frequency values were corrected with a scaling factor of 0.9608 taking into account a systematic error of the basis set [41]. In addition, the HOMO and LUMO frontier orbital surfaces and energies, molecular electrostatic potential (MEP) surface, and Mulliken atomic charges were calculated for the optimized structures at the same level of theory.

## 3. Results

### 3.1. Synthesis of the WSSL Sulfonic Derivatives

The main drawbacks of the sulfation of hydroxyl-containing organic compounds with sulfamic acid in the presence of urea are the impossibility of isolating urea from the reaction mass and the competing reaction of the carbamatization of hydroxyl groups [42,43,44,45].

In this work, we examined the effect of the type of a solid mesoporous acid catalyst on the degree of sulfation and the composition and structure of the products of WSSL sulfation with sulfamic acid. The catalysts used were Sibunit oxidized at 500˚C (granules, Sibunit-ox-500g), Sibunit oxidized at 400˚C (powder, Sibunit-ox-400), sulfonated Sibunit (Sulfated Sibunit), TiO_2_, and γ-Al_2_O_3_. The acidity of the catalysts was estimated using the PZC.

It is evident from the data in Table 1 that lignin sulfation of the aluminum γ-Al_2_O_3_ and titanium TiO_2_ oxides yields the WSSL derivatives with the lowest sulfur content (8.1 and 5.5 wt%, respectively); as the catalyst acidity increases, the degree of lignin sulfation decreases due to the competing polymer degradation processes. In addition, the aluminum and titanium oxides have fairly large sets of Lewis acid and base centers. The concentration and strength of these centers depends on the method used to obtain the corresponding hydroxides, the calcination temperature determining the crystal structure formed, and the nature of the chemical modifying agents [46].

It should be noted that the data obtained in this work are slightly different from the results reported in the studies on preparing the sulfo-derivatives of betulin [17,35], which may be due to the nature of the initial compounds and their different stabilities in the acid hydrolytic destruction processes [1,2,3].

In addition, as reported in [24], the Lewis acids used as the catalysts for WSSL sulfation lead to the production of WSSL sulfates with a sulfur content similar to that of the products of sulfation with sulfamic acid in the presence of some organic bases (e.g., thiourea, DMF, and pyridine).

It is noteworthy that the maximum sulfur content (16.5 wt%) in the synthesized WSSL derivatives noticeably exceeds the values reported earlier for the sulfation of lignins over base catalysts. In particular, when sulfating WSSL with sulfamic acid in the presence of urea in 1,4-dioxane, lignin sulfates with a maximum sulfur content of 10.1 wt% were obtained [24].

The use of Sibunit-4 modified under different conditions leads to the production of sulfated products with a higher degree of lignin sulfation compared to its value in the sulfated products obtained with the aluminum and titanium oxides. The maximum sulfur content (16.5 wt.%) in the WSSL-sulfated derivative was obtained from the Sib-unit-ox-400 graphite-like catalysts. When the Sibunit-ox-500 g granular graphite-like catalyst was used, the sulfur content in the products was lower (10.7 wt%) than in the case of the Sibunit-ox-400 powder catalyst, which can be explained by the diffusion limitations imposed on the delivery of reagents to the catalyst reaction sites. A similar effect of the difference between the catalyst surfaces on the sulfation process was described in [47], where granular catalysts were shown to be less active in the hydrogenation of flax shives.

The high degree of the sulfation of the catalysts based on the Sibunit-4 graphite-like material can be attributed to the fact that the reaction occurs both at the aliphatic and aromatic hydroxyl groups of the lignin macromolecule or to the growth in the number of hydroxyl groups available for sulfation, which appear during the depolymerization of lignin (Table 1). On the contrary, as was proven previously [26,28], the sulfation of lignin with sulfamic acid in the presence of urea is only accompanied by the selective sulfation of aliphatic hydroxyls of lignin without affecting the aromatic ones.

In the Brønsted acids (Table 1), we observed the following dependence of the sulfur content in the lignin sulfate on the catalyst acidity: the lower the pH_pzc_ value, the lower the sulfur content in the WSSL sulfo derivatives. Such a catalytic effect of the acid catalysts on the sulfation process may be due to the presence of both the acid and base centers on their surface. The carbonyl and ether groups and their combinations forming the pyrone structures in the Sibunit carbon material-based catalyst are known to be basic. Aromatic π-electrons in the graphite layers can also be responsible for the basic properties of carbon [48]. As the acidity decreases, the number of base centers on the catalyst surface obviously increases; this, in turn, leads to an increase in its activity in the sulfation process. According to the correlated dependences of the degree of lignin sulfation on the acid nature of the catalysts, the mechanism of their catalytic action is similar to the catalytic effect of some organic bases on the sulfation of natural compounds with sulfamic acid [24,26,28,48,49,50,51].

The proposed mechanism of the WSSL sulfation with sulfamic acid on the Sulfated Sibunit catalyst allows us to describe the process as

R–X^−^H^+^ + SO_3_∙NH_3_ → R–X^−^NH_4_^+^ + SO_3_ (1)

SO_3_ + 1,4-dioxane → SO_3_ *∙1,4-dioxane (2)

SO_3_ *∙1,4-dioxane (sulfating complex) + R′–OH → R′–O–SO_3_H + 1,4-dioxane (3)

R′–(O-SO_3_H)_2_ + R–X^−^NH_4_^+^ → R′–O-SO_3_ NH_4_ + R−X^−^H^+^, (4)

where R is the catalyst matrix, R′ is the WSSL molecule, and X^−^H^+^ is the catalyst functional group.

According to our reaction scheme proposed above, during the catalytic sulfation of WSSL, the zwitterionic form of sulfamic acid is first sorbed on the catalyst matrix, which is followed by its decomposition into sulfur trioxide and ammonia (the limiting stage) [52]. After that, sulfur trioxide reacts with the formation of a reactive sulfating complex with 1,4-dioxane, which in turn sulfates the WSSL molecule. Next, the catalyst matrix and the acidic form of WSSL sulfate exchange via ammonium cations. This scheme is similar to our scheme for the catalytic sulfation of betulin [17,35].

Thus, the acid-base properties of the solid catalyst surface govern the lignin sulfation process. Brønsted acids catalyze the modification of WSSL with sulfamic acid better than Lewis acids. The high degree of sulfation with the catalysts based on the Sibunit-4 graphite-like material can be related to the occurrence of the reaction both at the aliphatic and aromatic hydroxyl groups of the lignin macromolecule or to the growth in the number of hydroxyl groups available for sulfation as a result of the depolymerization of lignin (Table 1). When using the Sibunit-based catalysts, along with the sulfation process, similarly to the growth of the catalyst acidity, the lignin depolymerization processes are intensified, which causes the formation of sulfated lignin with the lowest weight average molecular weight (Table 1).

### 3.2. FTIR Study

**Figure 1:**
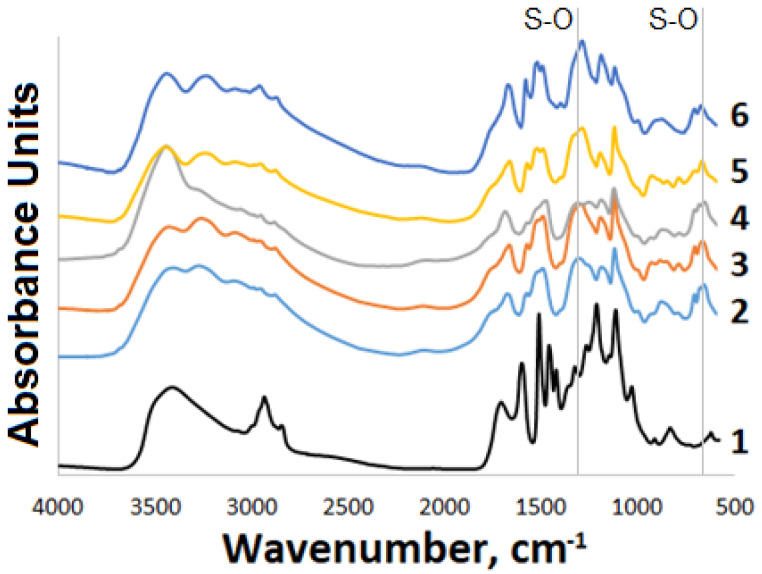
FTIR spectra of modified WSSL: (1) initial and obtained with (2) the Sibunit-ox-500 g, (3) Sibunit-ox-400, (4) Sulfated Sibunit, (5) TiO_2_, and (6) γ-Al_2_O_3_ catalysts.

The IR spectroscopy data on the initial and modified WSSL are presented in Figure 1. It is evident that the modified WSSL profile differs significantly from the profile of the initial WSSL. In particular, the spectrum of the modified WSSL contains absorption bands at 1255–1210, 840–790, and 590–690 cm^−1^, which are indicative of the presence of a sulfate group after the modification. In the region of 3500–2500 cm^−1^, the absorption bands of the hydroxyl, alkyl, and ammonium groups are superimposed, which causes the significant broadening of the peaks.

### 3.3. UV–Vis

**Figure 2:**
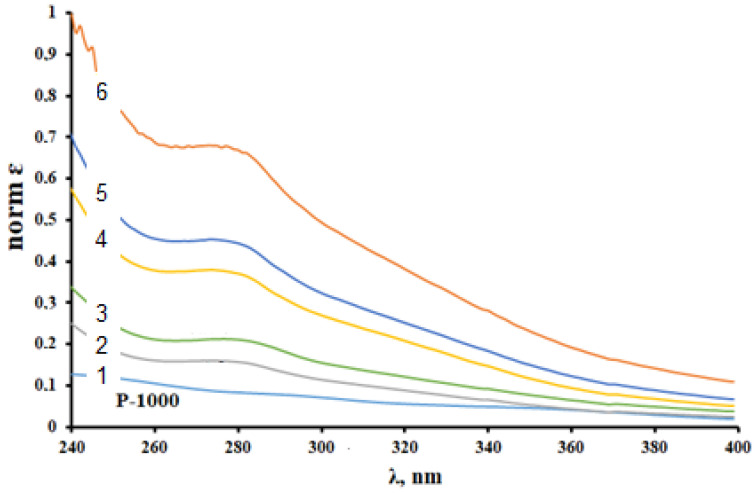
UV absorption spectra for (1) the initial WSSL, (2) Sulf. WSSL (Sib-ox-500g), (3) Sulf. WSSL (Sulf. Sibunit), (4) Sulf. WSSL (Sib-ox-400), (5) Sulf. WSSL (TiO_2_), and (6) Sulf. WSSL (Al_2_O_3_) lignin samples.

It is evident in the normalized UV absorption spectra (Figure 2) that the main peak characteristic of the alcoholic solutions of lignins (270–290 nm) change nonmonotonically with the change in the catalyst used. The maximum absorption at 270–290 nm is caused by the nonconjugated phenolic groups in lignin [30,31]. The most pronounced peak in this region corresponds to the Sulfated-Sibunit catalyst and can be explained by a larger amount of the nonconjugated phenolic groups in the macromolecule as a result of the more intense depolymerization of the initial lignin (Table 1). For the cat-3 catalyst, which ensures the formation of a sulfated product with the highest sulfur content, this peak is suppressed almost completely. This can be explained by a decrease in the absorption intensity of the phenolic groups due to their transition to the sulfated structure or by a significant change in the polymer structure, after which the free OH groups can no longer effectively absorb UV radiation.

### 3.4. GPC

**Figure 3:**
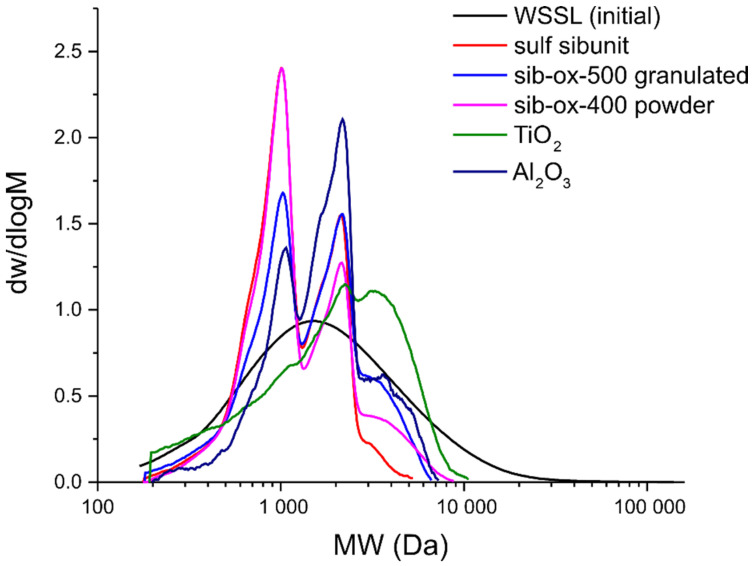
Molecular weight distribution for the WSSL samples.

The data shown in Figure 3 show that the initial WSSL is characterized by a monomodal distribution represented by polymers with molecular weights from ~200 to 20,000 Da [24]. The modification of lignin by the catalysts strongly changes its structure. We note that, in all the cases, the modification of WSSL reduces the polymer molecular weights (see Table 1), which is reflected in the shift of the molecular weight distribution (MWD) curves to the low molecular weight region.

The TiO_2_ catalyst has the weakest effect on the molecular weight of lignin; the MWD curve shifts slightly to the right due to an increase in the molecular weights of the individual polymer molecules caused by the incorporation of sulfate groups into their structure. The PDI of the sample decreases to 2.06 due to the removal of the low molecular weight fraction during dialysis.

In the case of the Al_2_O_3_ catalyst used, the MWD curve of the sulfated lignin derivative changes: the pronounced peaks corresponding to molecular weights of ~900 and ~2000 Da appear, which are probably related to the products of the partial depolymerization of the initial lignin macromolecules. At the same time, the weak peak around ~4000 Da corresponds to the structures that degraded the least during sulfation. At the same time, due to a decrease in the fraction of high molecular weight structures, the PDI of the sample decreased to 1.47.

When the Sibunit^®^-based catalysts are used, the fraction with *M*_w_ ~900 Da grows due to the intensified degradation of the lignin polymers. Moreover, the use of sulfated Sibunit^®^ and oxidized Sibunit^®^ yields a product with the MWD profiles identical in this region. The difference is in the almost complete absence of the polymer particles with an *M*_w_ value of more than 3000 Da in the sample. Therefore, this sample has the highest uniformity and polydispersity (1.34). In the presence of granular Sibunit^®^, probably due to the diffusion limitations, the depolymerization of lignin proceeds less intensively, the MWD curve contains a minor peak corresponding to the high molecular weight (3000 Da) structures, and the low molecular weight (900 Da) fraction is slightly larger than in the sample obtained using Al_2_O_3_.

### 3.5. TGA/DSC

**Figure 4:**
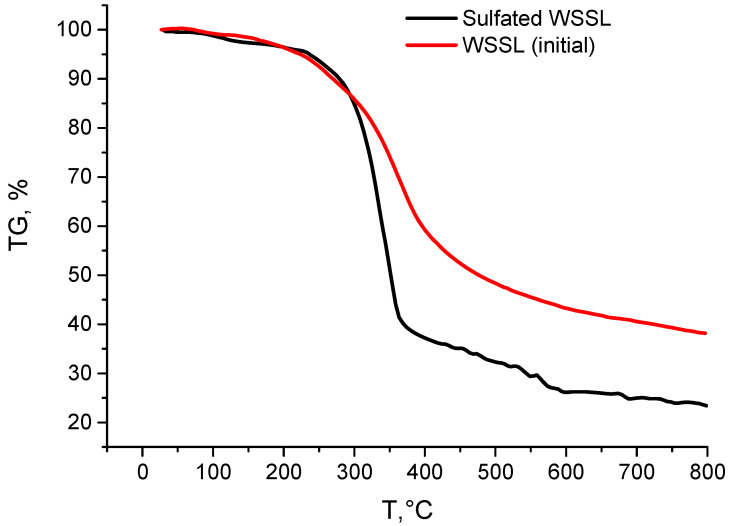
Results of the thermogravimetry study.

**Figure 5:**
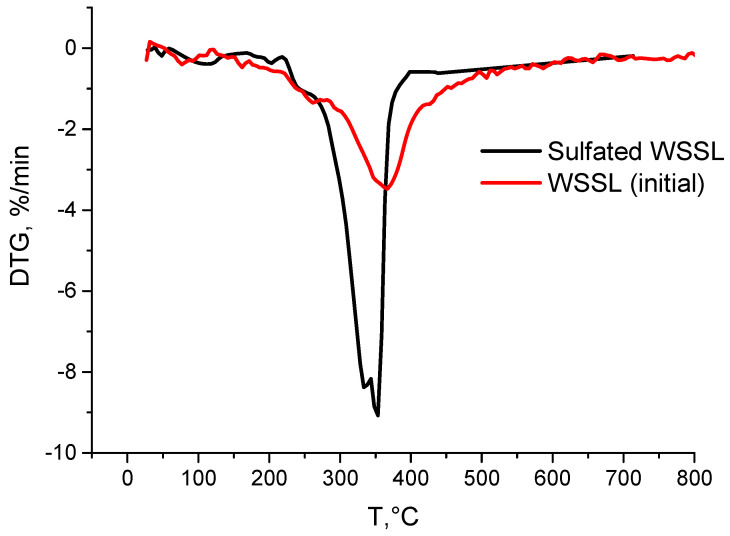
Differential thermogravimetry data.

The data of the thermal analysis are shown in Figure 4 and Figure 5.

The fractions of the substance decomposed upon heating from 250 °C at a rate of 10 deg/min determined from the thermograms of the initial and sulfated lignin samples are listed in Table 2. It is evident that the investigated lignins were generally decomposed in a wide temperature range by 700 °C. A comparison with the decomposition of the samples during thermolysis shows that the investigated lignins have different weight loss indicators at the same temperatures. Sulphated lignin demonstrates the maximum weight loss, and this trend continues until the pyrolysis is completed.

Both lignins are decomposed mainly in the range from 250–500 °C, i.e., in the active pyrolysis zone. Above 400 °C, a coke residue starts forming due to the aromatization of the lignin structure [53,54].

The final stage of passive pyrolysis is accompanied by weight loss, which was 76.6 and 61.9% for the initial and sulfated lignin, respectively, by 800 °C. The coke residue of the initial lignin at any stage of its formation is smaller than that of sulfated lignin, which may indirectly indicate a smaller number of thermostable fractions in its structure.

It is evident from the thermograms presented in Figure 5 that the DTG decomposition curve for the initial straw lignin sample has a broad peak, characteristic of lignins, with a shoulder in the range from 200 to ~340 °C. According to [55], in the temperature range of 230–260 °C, the propane side chains of lignin degrade with the formation of the methyl, ethyl, and vinyl derivatives of guaiacol. In addition, at temperatures of ≤310 °C, the thermally unstable ether bonds are destructed [56]. The thermogram of the sulfated lignin also contains a pronounced shoulder in the range from 200 to ~340 °C, but unlike the case of the initial lignin, it includes a narrow intense peak with a maximum at 350 °C, which is probably related, among other things, to the removal of the sulfo groups.

### 3.6. AFM

**Figure 6:**
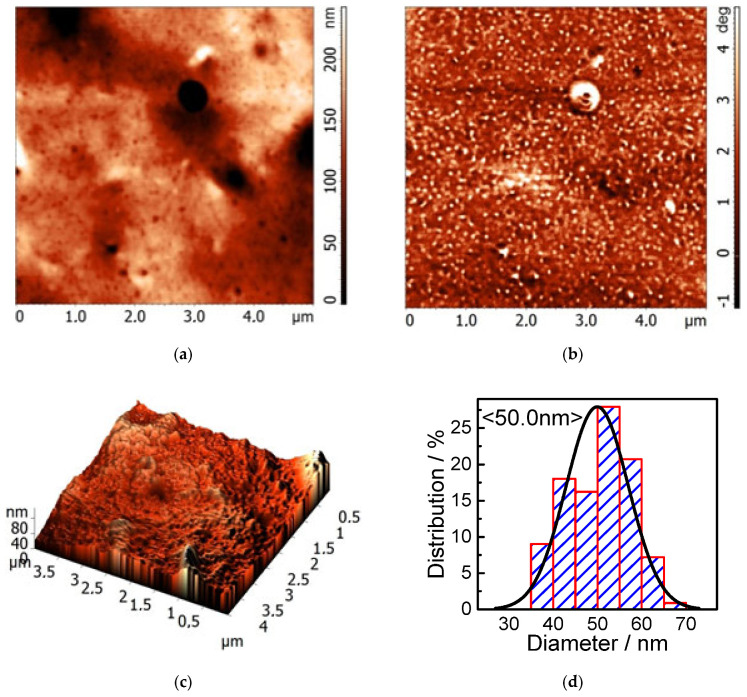
Atomic force microscopy data: (**a**) relief, (**b**) phase contrast, (**c**) 3D relief, and (**d**) particle size distribution diagram.

The sulphated WSSL films were studied by atomic force microscopy (AFM). The AFM images (Figure 6) show that the sulfated WSSL film surface consists of homogeneous particles about 50 nm in size that are uniformly distributed over the film’s surface without forming aggregates. It should be noted that, upon the sulfation of WSSL with sulfamic acid in the presence of urea, the particle size in the film was 100–200 nm [24].

The incorporation of functional (e.g., sulfate) groups into the polymer structure, which intensify the inter- and intramolecular interactions or polyelectrolyte effects [57,58,59], can enhance the aggregation and coagulation of a polymer. Since we obtained sulfated lignin with a higher (compared to the known [24]) sulfur content, and consequently a high content of sulfate functional groups, we can assume that in the investigated case, the coagulation of sulfated lignin occurs.

According to the phase–contrast image (Figure 6b), the film surface is homogeneous and contains no impurities.

The sulfated lignin particle size distribution (Figure 6d) is Gaussian.

### 3.7. DFT Analysis

#### 3.7.1. Structure Optimization and HOMO–LUMO Analysis

Lignin is a complex aromatic biopolymer, which complicates the DFT quantum-chemical calculation. The heterogeneous macromolecular structure of lignin is understudied. Therefore, the DFT calculation was focused upon several model compounds containing different bonds (β-O-4, α-O-4, β-β, β-1, 5-5′, etc.) [60,61,62,63].

**Figure 7:**
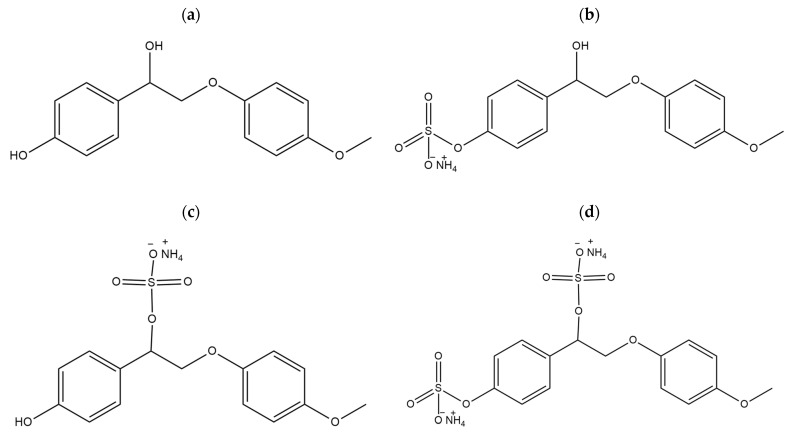
Investigated β-O-4 lignin molecules: initial β-O-4 lignin (**a**), and sulfated β-O-4 lignin with the sulfate group localized at the aromatic (**b**), alkyl (**c**), and alkyl and aromatic (**d**) hydroxo groups.

As a model compound for the quantum-chemical calculation of lignin and sulfated lignin, we chose β-O-4 lignin, specifically, 4-(1-hydroxy-2-(4-methoxyphenoxy)ethyl)phenol (Figure 7a). To study the effect of the sulfation mechanism on the physicochemical properties, the molar compounds of β-O-4 lignin with the sulfate group localized at the alkyl, aromatic, and alkyl and aromatic hydroxo groups were also calculated (Figure 7b–d). In [64], we investigated the quantum-chemical characteristics of sulfated monolignols and we continue our study herein. The theoretically investigated molecules of β-O-4 lignin and its sulfate are shown in Figure 7.

First, the structures under study were optimized to find the most energetically favorable conformations.

**Figure 8:**
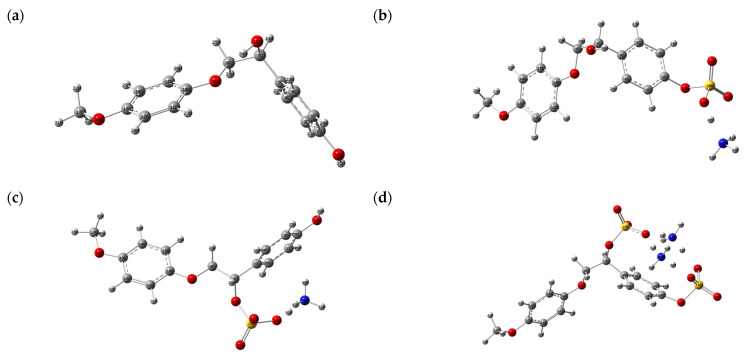
Optimized molecular structure: initial β-O-4 lignin (**a**), and sulfated β-O-4 lignin with the sulfate group localized at the aromatic (**b**), alkyl (**c**), and alkyl and aromatic (**d**) hydroxo groups.

The molecular geometry of the initial and sulfated β-O-4 lignin were optimized in the B3LYP/6-31G (d, p) basis set (Figure 8).

It is evident in Figure 8 that a sulfate group incorporated into the β-O-4 lignin molecule changes the conformation and configuration of the latter.

**Figure 9:**
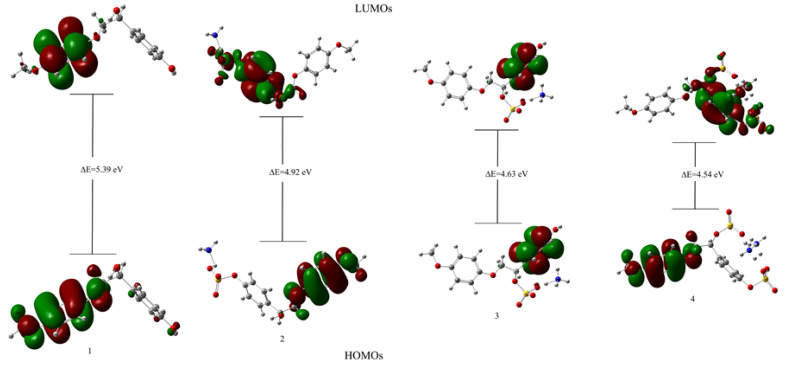
3D representation of the HOMO–LUMO orbitals and their energies for the molecules under study.

In the analysis of the charge transfer interactions, the highest occupied molecular orbital (HOMO) and the lowest unoccupied molecular orbital (LUMO) and the corresponding energies are of great importance [65]. The HOMO and LUMO frontier molecular orbitals of a molecular system are conventionally used to estimate its chemical reactivity [66,67]. The HOMO can donate an electron, while the LUMO is considered to be an electron acceptor due to its ability to gain an electron [65]. At the occurrence of a chemical reaction in a molecular system, electrons pass through the HOMO–LUMO gap due to the energy change [68]. The HOMO–LUMO energy gap is used to predict the chemical reactivity of molecular systems [69]. Generally, a molecular system with a large HOMO–LUMO gap is less reactive than a system with a smaller gap. The HOMO and LUMO of the compounds under study and their energy plots are presented in Figure 9.

The data of the HOMO–LUMO analysis (Figure 9) show that the initial β-O-4 lignin has the highest Δ*E* value (5.39 eV). The sulfation of the alkyl hydroxide reduces it to 4.92 eV and the sulfation of the aromatic hydroxyl group to Δ*E* = 4.63 eV. The lowest Δ*E* value (4.54 eV) is obtained upon substitution of both the aromatic and aliphatic hydroxyl groups into the β-O-4 lignin molecule.

Thus, the HOMO–LUMO analysis allowed us to assume that β-O-4 lignin sulfated to the aliphatic position will be more stable than that sulfated to the aromatic position and the β-O-4 lignin with the sulfate groups in the aliphatic and aromatic positions has the highest reactivity.

The HOMO and LUMO energies were used to the calculate electron affinity *A*, electronegativity χ, energy band gap Δ*E*, chemical potential μ, chemical hardness η, ionization energy *I*, softness ς, maximum charge transfer index Δ*N*_max_, nucleophilic index *N*, and electrophilicity index ω for the initial and sulfated β-O-4 lignin by the techniques described in [70]. The data obtained are given in Table 3.

According to the data listed in Table 3, the chemical potentials of the initial and sulfated β-O-4 lignin are negative, i.e., these molecules are stable. The chemical hardness refers to the resistance of a chemical system to the deformation of the electron cloud during chemical treatment [71]. Chemically hard systems with a large HOMO–LUMO energy gap are less polarizable and relatively small, while soft systems with a small HOMO–LUMO energy gap are more polarizable and large [72].

It is evident from Table 3 that the electron affinity *A* of the initial β-O-4 lignin has the lowest value (0.0963), while the maximum *A* value (0.9706) was observed for sulfated β-O-4 lignin with the aliphatic and aromatic sulfate groups. The chemical hardness η is at its maximum (2.6950) for the initial β-O-4 lignin and at its minimum (2.2701) for sulfated β-O-4 lignin with the aliphatic and aromatic sulfate groups, while the minimum softness ς (0.3711) among the investigated compounds corresponds to the initial β-O-4 lignin and the maximum softness (0.4405) pertains to the sulfated β-O-4 lignin with the aliphatic and aromatic sulfate groups. The minimum electronegativity χ (2.7913) was observed in the initial β-O-4 lignin, while the maximum value (3.2407) corresponds to sulfated β-O-4 lignin with the aliphatic and aromatic sulfate groups, which is caused by the introduction of a negatively contaminated sulfate group into the lignin molecule. This phenomenon is directly related to the change in the electrophilicity index ω from 1.4456 (for the initial β-O-4 lignin) to 2.3132 (for sulfated β-O-4 lignin with the aliphatic and aromatic sulfate groups). The situation is the opposite of the nucleophilic index *N*: it decreases from 0.6918 to 0.4323 at the transition from the initial β-O-4 lignin to sulfated β-O-4 lignin with the aliphatic and aromatic sulfate groups.

#### 3.7.2. MEP Analysis

The MEP data obtained by a robust quantum-chemical method are used to interpret and predict various aspects of the chemical reactivity [73,74]. The MEP is a real physical property, which can be determined by X-ray diffractometry [75] or rigorous calculations [76].

The MEP is conventionally visualized by building a surface that reflects the boundaries of molecules. This can be made by overlapping the van der Waals radii of all atoms in a molecule using the algorithms for calculating the surface of a molecule accessible to the solvent or using a constant electron density value [77].

The MEP maps (the electrostatic potential energy maps) illustrate the 3D charge distribution in the molecules and are used to determine the nature of intermolecular interactions [73]. Such maps help visualize the charge distribution and charge-related properties of molecules, as well as their size and shape. The analysis of the electrostatic potential is related to the electron density and used to establish the positions of the electrophilic and nucleophilic attacks and the interactions of hydrogen bonds [21,78,79].

**Figure 10:**
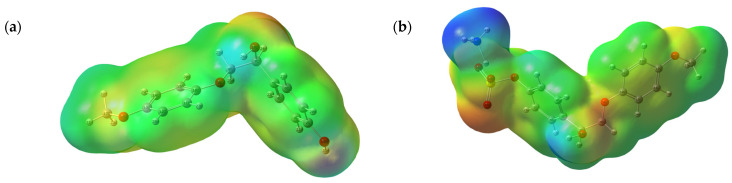

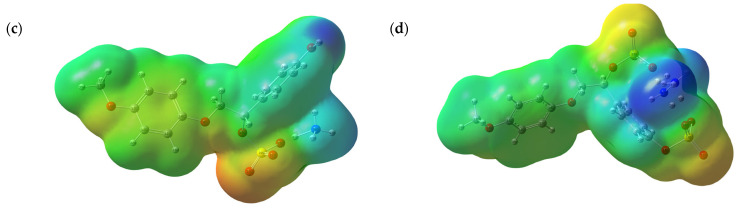
3D plot of the MEP surface for the investigated molecules: initial β-O-4 lignin (**a**), and sulfated β-O-4 lignin with the sulfate group localized at the aromatic (**b**), alkyl (**c**), and alkyl and aromatic (**d**) hydroxo groups.

Different colors in the MEP surface plot presented in Figure 10 correspond to different electrostatic potentials. The potential descending order is blue > green > yellow > orange > red. The negative values (colored red) correspond to the electrophilic attack region and are located mainly on an oxygen atom in the sulfate group. The nucleophilic attack (positive) region is colored blue and related mainly to hydrogen atoms in the ammonium cation.

#### 3.7.3. Mulliken Atomic Charge Analysis

Atomic charges and charge transfer are frequently used in the chemical determination of molecular behavior and reactivity. Atomic charges still play an important role in quantum chemistry and the concept of an atomic charge has been refined in many studies. Since the Mulliken’s description of atomic populations [80,81,82,83], many alternative definitions of atomic charges and populations have been presented [84]. The Mulliken charges are the total charges of atoms in a molecule, while the electrostatic potentials are generated by an electric field of the internal charge distribution [85,86]. The Mulliken atomic charges of the initial and sulfated β-O-4 lignin were calculated in the B3LYP/6-31 G(d, p) basis set.

The atomic charges of the initial and sulfated β-O-4 lignin obtained using the Mulliken population analysis are listed in Table 4.

The Mulliken atomic charges are related to the vibrational properties of a molecule and also affect the molecular polarizability, atomic charge effect, electronic structure, and many other properties of molecular systems [80,81,82,83].

The sulfate groups introduced into the β-O-4 lignin structure change the Mulliken charges of almost all the atoms (Table 4).

In particular, the charge observed for the 1C atom of the initial β-O-4 lignin is −0.1031, for lignin sulfated at the aliphatic hydroxyl it is −0.06686, for lignin sulfated at the aromatic hydroxyl it is −0.10693, and for lignin sulfated at both the aliphatic and aromatic hydroxyl groups the charges are both −0.10445. A somewhat different picture is observed for the 5C atom: for the initial sample, we have 0.10358; for sample 2, 0.08089; for sample 3, 0.06617; and for sample 4, 0.15057. The visible changes in the Mulliken atomic charges also affect the oxygen, hydrogen, nitrogen, and sulfur charges. Thus, the incorporated functional sulfate group influence the properties and charge of the entire β-O-4 lignin molecule.

#### 3.7.4. Theoretical FTIR Analysis

The density functional theory (DTF) [87,88] is widespread in the theoretical examination of the physicochemical characteristics of natural substances. Here, the calculation of spectroscopic properties is of great importance for predicting the relevant physicochemical characteristics [89,90,91,92].

**Figure 11:**
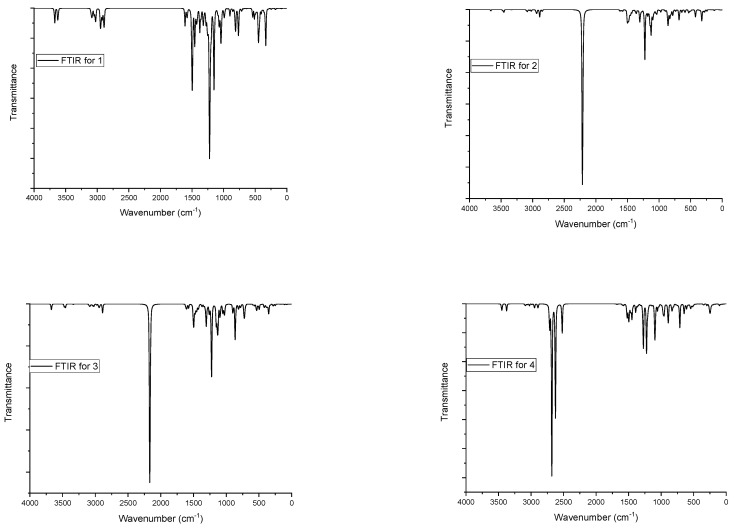
Theoretical FTIR spectra of the investigated molecules.

To refine the spectroscopic characteristics of the initial and sulfated β-O-4 lignins, a theoretical FTIR spectrum was calculated. The 6-31G (d, p) DFT FTIR spectra of the initial and sulfated β-O-4 lignins contain absorption bands with different relative intensities (see Figure 11 and Table 5).

O–H vibrations

In the theoretical FTIR spectra of the initial β-O-4 lignin, stretching vibrations of OH groups were observed around 3673 and 3623 cm^–1^, respectively. For sulfated β-O-4 lignin (samples 2 and 3), the OH-group stretching vibrations were observed at 3662 and 3670 cm^−1^, respectively. For lignin sulfated to the aliphatic and aromatic positions, no OH stretching vibrations were observed.

C–H vibrations

The aliphatic C–H group gives rise to several fundamental frequencies, including stretching (symmetric and asymmetric in-plane and out-of-plane bending vibrations). The asymmetric and symmetric tension modes appear, as a rule, at approximately 3100 and 3000 cm^–1^ [79,93]. In the theoretical FTIR spectra, vibrations of the aliphatic C–H group in the initial β-O-4 lignin were observed in the range of 3022–2891 cm^−1^. For sulfate β-O-4 lignin, the aliphatic C–H groups fluctuated in the range of 3020–2848 cm^−1^. For the aromatic C–H groups in the initial β-O-4 lignin, the fluctuations were observed at 3101–3034 cm^−1^ and for sulfated β-O-4 lignin, at 3099–3052 cm^−1^.

O–S vibrations

In the theoretical FTIR spectra, stretching vibrations of the O–S group for lignin sulfated at the aliphatic hydroxyl group β-O-4 lignin were observed at 1302, 1101, 858 cm^−1^ at 1303, 1098, and 861 cm^−1^, and for lignin sulfated at both the aliphatic and aromatic positions, the absorption bands were observed at 1276, 1272, 1095, 1087, 955, and 890 cm^−1^. In the experimental FTIR spectra of the sulfated natural substances, the vibration of this group was observed at 1249–1259 and 815–850 cm^–1^, which is consistent with the results reported in [94,95].

C=C vibrations

In the theoretical FTIR spectra, C=C stretching vibrations for the initial and sulfated β-O-4 lignin were observed at 1611–1568 cm^−1^.

N–H vibrations

Vibrations of N–H groups were observed in the range of 3452–3336 cm^–1^ only in β-O-4 lignin sulfated at different positions due to the presence of an ammonium cation.

## 4. Conclusions

First, this research proposed to carry out the sulfation of wheat straw soda lignin with sulfamic acid over solid catalysts of different types.

Then, it was demonstrated that the maximum sulfur content (16.5 wt%) can be obtained with the Sibunit-4^®^ catalyst oxidized at 400 °C, while the Lewis acids (aluminum and titanium oxides) appeared to be less efficient.

The incorporation of a sulfate group was proven by the elemental analysis and FTIR spectroscopy. For the WSSL sulfates, in contrast to the initial WSSL, the absorption bands in the region of 1255–1210 cm^−1^ were observed, which correspond to the sulfate group’s vibration.

Using GPC, it was found that the solid catalysts used in the sulfation process cause hydrolysis reactions and reduce the molecular weight and polydispersity index.

The characteristics of the model compounds of the initial and sulfated β-O-4 lignin were calculated within the density functional theory using the MEP, HOMO–LUMO, and Mulliken atomic charge analysis.

## Figures and Tables

**Table 1:** Effect of the catalysts on the sulfur content, weight average molecular weight *M*_w_, and number of average molecular weight *M*_n_ of the WSSL derivatives (the solvent is 1,4-dioxane).

№	Catalyst	pH_pzc_	Sulfur Content, wt.%	*M*_n_ (g/mol)	*M*_w_ (g/mol)	PDI
1	WSSL (initial)	-	-	1067	2779	2.60
2	Sibunit-ox-500 g	3.34	10.7	1066	1680	1.58
3	Sulfated Sibunit	4.26	12.2	961	1290	1.34
4	Sibunit-ox-400	6.88	16.5	1024	1536	1.50
5	TiO_2_	3.75	8.1	1175	2424	2.06
6	γ-Al_2_O_3_	6.71	5.5	1328	1946	1.47

**Table 2:** Main ranges of thermal decomposition of lignins upon heating at a rate of 10 deg/min.

Sample	Weight Loss (%) at Different Temperatures (°C)												
	200	250	300	350	400	450	500	550	600	650	700	750	800
Initial	3.7	7.7	14.4	26.4	41.1	47.7	51.7	54.6	56.8	58.3	59.5	60.8	61.9
Sulf.	3.5	6.2	14.7	47.0	62.7	64.9	67.6	70.6	73.9	74.0	75.0	75.9	76.6

**Table 3:** Electronic properties of the molecules under study.

Parameters	Values (eV)			
	1	2	3	4
*E*_LUMO_	−0.0963	−0.3720	−0.6855	−0.9706
*E*_HOMO_	−5.4864	−5.2970	−5.3157	−5.5109
Energy gap (Δ*E*)	5.3900	4.9250	4.6303	4.5402
Electron affinity (*A*)	0.0963	0.3720	0.6855	0.9706
Ionization energy (*I*)	5.4864	5.2970	5.3157	5.5109
Chemical hardness (η)	2.6950	2.4625	2.3151	2.2701
Softness (ς)	0.3711	0.4061	0.4319	0.4405
Chemical potential (μ)	−2.7913	−2.8345	−3.0006	−3.2407
Electronegativity(χ)	2.7913	2.8345	3.0006	3.2407
Electrophilicity index (ω)	1.4456	1.6313	1.9445	2.3132
Nucleophilic index (*N*)	0.6918	0.6130	0.5143	0.4323
Maximum charge transfer index (Δ*N*_max_)	1.0357	1.1511	1.2961	1.4276

Δ*E* = *E*_LUMO_ − *E*_HOMO_ (eV), *I* = −*E*_HOMO_ (eV), *A* = −*E*_LUMO_ (eV), χ = (*I* + *A*)/2 (eV), η = (*I* − *A*)/2 (eV), µ = −(*I* + *A*)/2 (eV), ω = µ^2^/2η (eV), ς = 1/2η (eV), *N* = 1/ω (eV), Δ*N*_max_ = −µ/η (eV).

**Table 4:** Mulliken atomic charges for the investigated molecules.

1		2		3		4	
Atoms	Charges	Atoms	Charges	Atoms	Charges	Atoms	Charges
1C	−0.1031	1C	−0.06686	1C	−0.10693	1C	−0.10445
2C	0.32776	2C	0.29177	2C	0.32407	2C	0.29368
3C	−0.13513	3C	−0.0774	3C	−0.15736	3C	−0.11439
4C	−0.12606	4C	−0.11321	4C	−0.13562	4C	−0.13892
5C	0.10358	5C	0.08089	5C	0.06617	5C	0.15057
6C	−0.12338	6C	−0.13989	6C	−0.10756	6C	−0.1529
7C	0.09773	7C	0.09447	7C	0.06414	7C	0.0546
8C	0.05415	8C	0.0841	8C	0.08727	8C	0.06606
9O	−0.55423	9O	−0.53056	9O	−0.53231	9O	−0.54192
10C	0.3519	10C	0.35404	10C	0.3479	10C	0.34901
11C	−0.12122	11C	−0.12083	11C	−0.11998	11C	−0.12101
12C	−0.11696	12C	−0.11686	12C	−0.11715	12C	−0.11679
13C	0.34825	13C	0.34628	13C	0.34699	13C	0.3484
14C	−0.14573	14C	−0.14569	14C	−0.14635	14C	−0.14668
15C	−0.13894	15C	−0.14184	15C	−0.14201	15C	−0.14166
16O	−0.52236	16O	−0.52371	16O	−0.52277	16O	−0.5222
17C	−0.07609	17C	−0.0748	17C	−0.07472	17C	−0.0762
18O	−0.55195	18O	−0.53871	18O	−0.55114	18O	−0.59512
19O	−0.56026	19O	−0.59243	19O	−0.54111	19S	1.31849
20H	0.09417	20S	1.31194	20S	1.32458	20O	−0.6431
21H	0.07219	21O	−0.53093	21O	−0.57469	21O	−0.48016
22H	0.07484	22O	−0.49368	22O	−0.46523	22O	−0.56656
23H	0.09159	23O	−0.59405	23O	−0.59541	23S	1.31134
24H	0.11311	24H	0.11232	24H	0.10842	24O	−0.59864
25H	0.10796	25H	0.09538	25H	0.08342	25O	−0.48728
26H	0.09915	26H	0.11256	26H	0.09894	26O	−0.63249
27H	0.09508	27H	0.08687	27H	0.12285	27O	−0.71167
28H	0.09733	28H	0.08226	28H	0.14238	28H	0.11397
29H	0.08569	29H	0.11075	29H	0.10135	29H	0.10509
30H	0.08744	30H	0.09728	30H	0.11362	30H	0.08854
31H	0.12344	31H	0.0926	31H	0.10314	31H	0.10441
32H	0.10908	32H	0.09329	32H	0.09634	32H	0.14052
33H	0.10877	33H	0.08342	33H	0.08311	33H	0.10006
34H	0.31644	34H	0.08511	34H	0.08448	34H	0.12339
35H	0.31571	35H	0.12139	35H	0.12192	35H	0.09645
		36H	0.10809	36H	0.10677	36H	0.09751
		37H	0.10781	37H	0.10856	37H	0.08618
		38H	0.30859	38H	0.31997	38H	0.08998
		39N	−0.76959	39N	−0.77406	39N	0.12368
		40H	0.42605	40H	0.2893	40H	0.10839
		41H	0.31383	41H	0.43481	41H	0.10962
		42H	0.28564	42H	0.28011	42N	−0.68977
		43H	0.28433	43H	0.30379	43H	0.39359
						44H	0.3195
						45H	0.39914
						46H	0.33304
						47N	−0.69366
						48H	0.3203
						49H	0.322
						50H	0.40576
						51H	0.40227

**Table 5:** Some important wavenumbers (cm^−1^) scaled by a factor of 0.9608 for the investigated molecules.

1		2		3		4	
O–H	3673 and 3623	ʋO–H	3662	ʋO–H	3670	ʋN–H	3452–3373
ʋC–H (aromatic)	3101–3034	ʋN–H	3475–3334	ʋN–H	3474–3336	ʋC–H (aromatic)	3099–3052
ʋC–H (aliphatic)	3022–2891	ʋC–H (aromatic)	3101–3052	ʋC–H (aromatic)	3100–3040	ʋC–H (aliphatic)	3022–2891
ʋC=C	1612–1574	ʋC–H (aliphatic)	3020–2848	ʋC–H (aliphatic)	3021–2887	ʋC=C	1611–1568
ʋO–C	1254–1220	ʋC=C	1612–1573	028BC=C	1612–1574	ʋO–C	1241 and 1223
		ʋO–C	1242 and 1224	ʋO–C	1239 and 1223	ʋS–O	1276, 1272, 1095, 1087, 955, 890
		ʋS–O	1302, 1101, 858	ʋS–O	1303, 1098, 861		

## Data Availability

Not applicable.
